# Health outcomes measurement and organizational readiness support quality improvement: a systematic review

**DOI:** 10.1186/s12913-018-3828-9

**Published:** 2018-12-29

**Authors:** Nynke A. Kampstra, Nina Zipfel, Paul B. van der Nat, Gert P. Westert, Philip J. van der Wees, A. Stef Groenewoud

**Affiliations:** 1 Department of Value-Based Healthcare, St. Antonius Hospital, Nieuwegein, the Netherlands; 2 Radboud Institute for Health Sciences, Scientific Center for Quality of Healthcare (IQ healthcare), Radboud university medical center, Nijmegen, the Netherlands

**Keywords:** Clinical registries, Quality improvement, Value-based healthcare, Improvement science

## Abstract

**Background:**

Using outcome measures to advance healthcare continues to be of widespread interest. The goal is to summarize the results of studies which use outcome measures from clinical registries to implement and monitor QI initiatives. The second objective is to identify a) facilitators and/or barriers that contribute to the realization of QI efforts, and b) how outcomes are being used as a catalyst to change outcomes over time.

**Methods:**

We searched the PubMed, EMBASE and Cochrane databases for relevant articles published between January 1995 and March 2017. We used a standardized data abstraction form. Studies were included when the following three criteria were fulfilled: 1) they relied on structural data collection, 2) when a structural and comprehensive QI intervention had been implemented and evaluated, and 3) impact on improving clinical and/or patient-reported outcomes was described. Data on QI strategies, QI initiatives and the impact on outcomes was extracted using standardized assessment tools.

**Results:**

We included 21 articles, of which eight showed statistically significant improvements on outcomes using data from clinical registries. Out of these eight studies, the Chronic Care Model, IT application as feedback, benchmarking and the Collaborative Care Model were used as QI methods. Encouraging trends in realizing improved outcomes through QI initiatives were observed, ranging from improving teamwork, implementation of clinical guidelines, implementation of physician alerts and development of a decision support system. Facilitators for implementing QI initiatives included a high quality database, audits, frequent reporting and feedback, patient involvement, communication, standardization, engagement, and leadership.

**Conclusion:**

This review suggests that outcomes collected in clinical registries are supportive to realize QI initiatives. Organizational readiness and an active approach are key in achieving improved outcomes.

**Electronic supplementary material:**

The online version of this article (10.1186/s12913-018-3828-9) contains supplementary material, which is available to authorized users.

## Background

The use of clinical registries is considered crucial to systematically measure clinical outcomes in achieving better value for patients [1]. A clinical or patient registry is defined as “an organized system that uses observational study methods to collect uniform data (clinical data as structure, process and outcome measures) to evaluate specified outcomes for a population defined by a particular disease, condition, or exposure” [2]. Registries that are used for evaluating patient outcomes are used for the purpose of this review. The importance of clinical registries has been widely recognized as a tool to realize quality improvement (QI) and public accountability [1, 3–8]. Medical associations use clinical registries for collecting data using pre-defined measures in patients undergoing a certain procedure or for a specific disease [9]. In particular, feedback based on clinical registry data is used to identify and monitor improvement initiatives [10]. Therefore, registries are seen as a promising tool to achieve improvements in value for the patient by measuring outcomes [1]. A previous review on the structure, use and limitations of current clinical registries showed that registries and their respective measures are used for monitoring the work of health care providers, discussion platforms for QI, improving risk adjustment modelling and for improving preoperative risk profiling [11]. However, the current body of literature lacks insights into the extent to which the use of outcome measures from clinical registries, either when identifying, selecting or monitoring QI initiatives, can impact health outcomes.

With rising healthcare costs, service restrictions, differences in quality and costs, there is an increasing need for reform to improve value of healthcare [12]. Value in healthcare is defined as outcomes relative to costs [13]. Value-based health care aims at achieving higher value for patients while ensuring sustainability of the healthcare system by an efficient and effective delivery of care [14]. This goal is assumed to be achieved by measuring and using outcomes per medical condition for the identification of improvement potential across the full cycle of care [12]. Higher value for patients by measuring outcomes is one of the potential methods for improving quality of healthcare relative to the costs spent. For the purposes of this review, we only focused on outcome measures and not on the respective costs.

Quality of healthcare is generally assessed by using structure, process or outcome measures [15]. The latter provide insights into outcomes of a certain disease or several diseases, for instance on survival, functional status, and quality of life [16]. The aim of measuring outcomes is diverse; guiding clinical decision-making, initiating improvement interventions, benchmarking, monitoring, scientific research and public accountability. Measuring outcomes structurally and using them to identify possible improvements contributes to the aim of achieving higher value for patients [17].

The goal is to summarize the results of studies which use outcome measures from clinical registries to implement and monitor QI initiatives. For the purpose of this study, QI was defined as the application of a defined improvement process to achieve measurable improvement by implementing an improvement intervention. Registry data itself is not sufficient as they need QI methods in order to achieve actual improvement. The second objective is to identify a) facilitators and/or barriers that contribute to the realization of QI efforts, and b) how outcomes are being used as a catalyst to change outcomes over time.

## Methods

A systematic review was conducted of studies published between January 1995 and March 2017. The search strategy was designed for PubMed, EMBASE and Cochrane databases. To identify evidence for the use of clinical registries to improve or contribute to patient health outcomes, the following PubMed Mesh terms were used to identify studies: *mortality*, *patient outcome assessment* and *treatment outcome*. These terms were combined with a variety of search terms related to QI and diverse disease specific registry studies. No specific patient group or study design was defined. Details of the complete search strategy are provided in the online supplementary content (Additional file 1: Appendix 1). Additional hand-searching has been conducted for systematic reviews on the subject during the review process. The hand-search was conducted in Google Scholar.

### Inclusion and exclusion criteria

Studies were included when they met each of the following criteria: 1) published in peer-reviewed journals, 2) published in English, French or German, 3) the study actively implemented a strategy using outcome data to realize QI, 4) the study relied on structural data collection, and 5) the study evaluated the QI interventions realized. Whether a study made use of a QI effort, falling under criteria 3 and 5, was evaluated after reviewing the full text papers and was therefore not part of the search string. After title screening, included studies were evaluated on criteria 3 and 5. Studies were excluded when they analyzed the effect of new intervention(s) on outcomes (testing drugs, new techniques or the effect of an intervention) or when the data had solely been collected to evaluate an intervention in a clinical trial.

### Data extraction and quality assessment

For the initial selection each reviewer reviewed a random set on first title, second abstract, and finally full text to determine eligibility. The full text articles were critically reviewed and judged by all reviewers. Any disagreement between reviewers was discussed by the full review team until consensus was achieved. The selected articles were evaluated using a standardized predesigned form listing whether the inclusion criteria were met.

A thorough review process was carried out for the data quality assessment, which consisted of the following three steps:

Step 1: Data abstraction

The Cochrane data abstraction form for intervention reviews (RCTs and non-RCTs) was used as a tool to extract data on study design and methodological quality (Additional file 1: Appendix 2) [18]. Furthermore, data on the target group, main results, main outcome measures, data source, geographical setting and funding sources was abstracted.

Step 2: Rigor of QI intervention

The included studies were evaluated using the Quality Improvement Minimum Quality Criteria Set (QI-MQCS) as a critical appraisal instrument, developed by the RAND Corporation (Additional file 1: Appendix 3) [19]. The QI-MQCS contains 16 domains to evaluate the QI intervention, resulting in a scoring system to evaluate whether this domain was met or not. The QI-MQSC did not introduce a threshold concerning acceptability of the quality of the papers. Therefore, we agreed on the following criteria in order to adequately interpret the QI-MQSC score. The study was considered to be of perfect quality (> 15 items ranked *yes*), good quality (> 12 items ranked *yes*), moderate quality (> 9 items ranked *yes*) and insufficient quality (≤9 items ranked *yes*).

Step 3: Rigor of data collection and analysis

In addition to the QI-MQCS, 13 items were added for further evaluation. Two questions (item 2 and 18) from the Downs & Black (1998) criteria were used to reflect on whether the main outcomes to be measured had been clearly described in the introduction or methods section and whether the statistical tests used to assess the main outcomes were appropriate [20]. In addition, three questions (item 10c, 11a and 11b) from the SQUIRE guidelines were used: 1) whether a method was employed for assessing completeness and accuracy of data, 2) whether quantitative methods were used to draw inferences from the data and 3) whether methods were applied for understanding variation within the data, including the effects of time as a variable [21]. Furthermore, it was evaluated how the included studies dealt with missing values, whether they performed audits, reported on secular trends, performed case-mix adjustments, whether clear inclusion and exclusion criteria had been defined for the patient population and when possible whether a power analysis was conducted.

In conclusion, the Cochrane data abstraction form was used to abstract data from the selected articles in order to identify changes in outcomes and facilitators. Data synthesis was guided by 1) the QI-MQCS results, 2) the merged and modified version of the Downs & Black (1998), SQUIRE guidelines, and self-developed questions. Due to the diversity of outcomes, a pooled effect of the results was not conducted.

## Results

### Search results and included studies

**Fig. 1:**
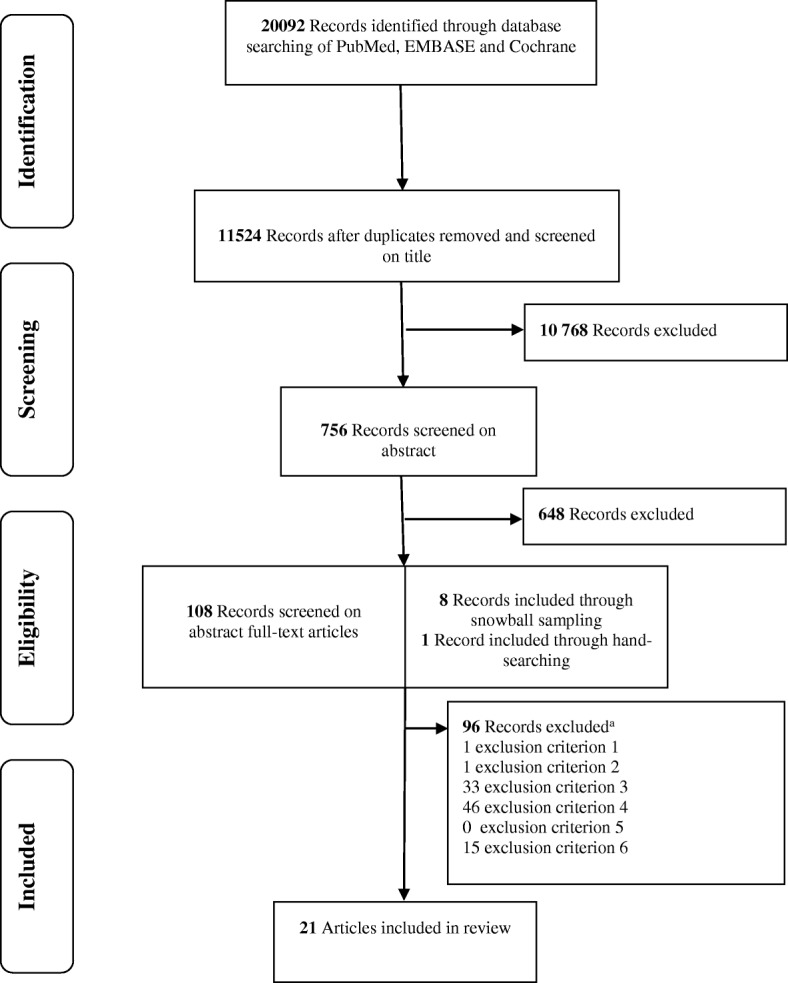
Flow diagram. Source: Authors’ analysis, format source from PRISMA [22]. ^a^ Exclusion criteria: 1. Studies published in peer-reviewed journals; 2. Studies published in English; 3. Did not actively implement a strategy making use of outcome data to realize quality improvement; 3. Did not relay on structural data collection; 5. Did not evaluate quality improvement interventions using data from outcome registries

The final systematic search resulted in 11 524 records for initial screening; 117 articles were included to review the full text version of which 96 studies were excluded because they did not meet the inclusion criteria (Fig. 1) [22]. One additional article was included from a relevant systematic review, which emerged from hand-searching [23, 24]. Table 1 presents the characteristics of the 21 included studies. The studies focused on registries for the following patient groups; patients with diabetes [24–31], children with chronic conditions [32], patients with lung cancer [33, 34], patients with cystic fibrosis [35–37], patients with cardiac anomalies [38], patients undergoing cardiac surgery [39–41], patients with acute myocardial infarction [42], and patients referred for home health services [43]. The majority of the registries presented voluntary participation [25–27, 29–31, 35, 36, 38, 40–43]. Three registries required mandatory participation [28, 33, 34]. Most of the presented registries had the purpose of achieving QI [24, 25, 28–34, 37, 39, 41–43]. The remaining studies have introduced their clinical registry for research and educational purposes [26, 27, 35, 36, 38, 40, 44].

**Table 1:** Characteristics of Included Studies (*n* = 21)

Characteristics	No. (%)
Geographical setting	
United States [24, 25, 27–29, 31, 32, 35, 37–41, 43, 44]	15 (71%)
Sweden [26, 42]	2 (9.5%)
Denmark [33, 34]	2 (9.5%)
Germany [36]	1 (4.8%)
Singapore [30]	1 (4.8%)
Target group	
Diabetes [24–31]	8 (38.1%)
Depression [44]	1 (4.8%)
Children with chronic conditions [32]	1 (4.8%)
Lung Cancer [33, 34]	2 (9.5%)
Cystic fibrosis [35–37]	3 (14.3%)
Congenital heart disease [38]	1 (4.8%)
Myocardial infarction [42]	1 (4.8%)
Patients undergoing cardiac or cardiothoracic surgery [39–41]	3 (14.3%)
Patients referred for home health services [43]	1 (4.8%)
Study design	
Observational study [29–31, 33–37, 39, 41]	10 (47.6%)
Randomized-Controlled Trial [24, 25, 27]	3 (14.3%)
Case study [26, 28]	2 (9.5%)
Cohort study [38]	1 (4.8%)
Before and after study [32, 40, 42]	3 (14.3%)
Quasi-experimental study [44]	1 (4.8%)
Prospective evaluation study [43]	1 (4.8%)
Funding sources	
National funding [25, 27, 28, 31, 32, 36, 42, 43]	8 (38.1%)
Private funding [24, 29, 35, 37, 44]	5 (23.8%)
Unknown [26, 30, 33, 34, 38–41]	8 (38.1%)
Registry participation type	
Voluntary [25–27, 29–31, 35, 36, 38, 40–43]	13 (62%)
Mandatory [28, 33, 34]	3 (14.3%)
Unknown [24, 32, 37, 39, 44]	5 (23.8%)
Registry purpose	
Quality improvement [24, 25, 28–34, 37, 39, 41–43]	14 (66.7%)
Research and education [26, 27, 35, 36, 38, 40, 44]	7 (33.3%)
Quality improvement efforts	
Benchmarking [33, 34, 38–41]	6 (28.6%)
Plan-do-check-act (PDCA) [26, 36]	2 (9.5%)
Collaborative Care Model [26, 28, 42, 44]	4 (19%)
The Chronic Care Model [25, 32]	2 (9.5%)
Learning and Leadership Collaborative [35]	1 (4.8%)
Plan-do-check-act (PDCA) and the Chronic Care Model [37]	1 (4.8%)
IT application as feedback tool [24, 27, 30, 41]	4 (19%)
No clear QI method [29, 31, 43]	3 (14.3%)

### Impact of quality improvement

Eight studies showed statistically significant improvement in outcomes resulting from the implementation of QI initiatives [25, 27, 29, 31, 33, 34, 42, 44]. Statistically significant improvements were achieved in long-term survival [33, 34], mortality [42], readmission rate [42], bleeding complications [42], systolic blood pressure [27], HbA1C [27, 29], LDL [27, 29], exercise habits [25], depression improved in the acute phase (PHQ-9 score) [44], and hospitalization with ambulatory care-sensitive conditions [31]. The remaining studies did not show statistically significant improvements. All included studies presented outcome measures for their respective improvement work, five of which also measured additional process measures [27–33, 35, 41, 42, 44]. Table 2 presents outcomes measures used, QI methods applied and whether statistically significant improvement of outcome measures was achieved. A detailed overview of the significance of outcome measures can be found in the online supplementary content (Additional file 1: Appendix 4). None of the studies identified an impact on patient value or evaluated the impact on costs of care.

**Table 2:** Improvement in outcomes and/or processes

Author/year	Outcome measures	Significant improvement + ^1^/0^2^/0^a 3^	RAND QI-MQCS score	QI methods	QI focus
Dziuban et al., (1994) [39]	Risk adjusted mortality	0^a^	12	Benchmarking	Hospital-specific and physician-specific results published annually in cardiac surgery.
Adams et al., (1998) [43]	Ambulation/locomotion	0	10	No clear QI method	Implementation of and outcome-based quality improvement concept including two outcome reports.
	Bathing	0			
	Management of oral medications	0			
	Pain	0			
	Dyspnoea	0			
Halpin et al., (2004) [40]	Postoperative Atrial fibrillation	0^a^	11	Benchmarking	Implementation of a new guideline based on insights into outcomes, literature and roundtable discussion. An Outcome Center was formed and a multidisciplinary Performance Improvement Committee.
	Operative mortality	0			
	Cardiac arrest	0			
	Reoperation for bleeding	0			
	Pneumonia	0			
	Deep sternal infection	0			
	Permanent stroke	0			
	Transient stroke	0			
	Prolonged ventilation	0			
	Length of stay	0			
Moller et al., (2005) [38]	Overall operative Mortality	0^a^	7	Benchmarking	Developed a centralized data acquisition and analysis method (through the creation of the network paediatric Cardiac Care Consortium). A uniform diagnostic and procedure classification system was created. Differences in patient populations cared for at the cardiac centres were compared.
Thomas et al., (2007) [24]	HgbA1c	0	13	IT application as feedback tool	Registry-generated audit, feedback and patient reminder targeted at residents.
	LDL cholesterol	0			
	Blood pressure	0			
Peterson et al., (2008) [27]	Mean systolic blood pressure	+	10	IT application as feedback tool	Multicomponent intervention: implementation of an electronic diabetes registry, visit reminders, and patient-specific physician alerts.
	HbA1c	+			
	Mean LDL	+			
Carlhed et al., (2009) [42]	Mortality	+	12	Collaborative Care Model	Multidisciplinary teams consisted of critical care unit nurses and cardiologists were assigned at each of the 19 volunteering hospitals. 19 teams of 4 to 5 persons met at 4 (group A) or 2 (group B) training sessions during which education by QI experts was provided, using the Breakthrough Series curricula.
	Readmission rate	+			
	Bleeding complication	+			
Jakobsen et al., (2009) [33]	1-year survival	+	5	Benchmarking	Indicators (staging, surgical procedures, complications and survival) have been registered in 5007 patients who underwent surgery. Each year the results have been audited locally, regionally and nationally and improvements have been proposed, implemented, monitored and evaluated by the audit-plenary.
	2-year survival	+			
	5-year survival	0			
	30-day mortality	0^a^			
Kraynack et al., (2009) [35]	FEV1	0^a^	12	Learning and Leadership Collaborative	A QI process is described from the initial team-building phase, through the assessment of care processes, standardization of care, and developing a culture of continuous improvement aiming to improve pulmonary function of the paediatric patients.
MacLean et al., (2009) [25]	Blood pressure	0	10	The Chronic Care Model	Providing decision support and patient decision support in diabetes care delivery.
	BMI	0			
	SF-12 Physical	0^a^			
	SF-12 Mental Quality of life	0			
	Exercise habit	+			
Toh et al., (2009) [30]	Poor HbA1c (9% and above)	0^a^	11	IT application as feedback tool	Chronic disease management system with patient reminders based on registry data.
	Good LDL-control below 2.6 mmol/L	0^a^			
Baty et al., (2010) [29]	% with HbA1c < 7%	+	10	No clear QI method	Implementing a comprehensive system-based disease management process including a diabetes registry and quality reports.
	% with HbA1c < 9%	+			
	%with LDL < 100	+			
Beaulieau et al., (2010) [41]	Mortality	0^a^	7	Benchmarking IT application as feedback tool	Implementing a method for linking administrative and registry data to track quality improvement initiatives through dashboards.
	Infusion rate	0^a^			
Bricker et al., (2010) [28]	A1C	0^a^	9	Collaborative Care Model	Implementing the Chronic Care Model through regional care learning collaborative with focus on team-based care, patient-centred care coordination, delivery of evidence-based care, patient self-management, use of a patient registry system and culturally and linguistically competent care.
	Blood pressure	0^a^			
	LDL Cholesterol levels	0^a^			
Bauer et al. (2011) [44]	Depression improved in acute phase (PHQ-9 score)	+	12	Collaborative Care Model	Implementing a collaborative care model including a web-based disease registry, care management to support treatment and organized psychiatric consultation.
	A1C testing	0	10	Plan-do-check-act (PDCA)	Realizing continuous quality improvement through benchmarking in cystic fibrosis care.
Stern et al., (2011) [36]	FEV1 > 80 < 18	0^a^			
	FEV1 > 80 > 18	0^a^			
	BMI > 19	0^a^			
	WH > 90	0^a^			
Jakobsen et al., (2013) [34]	1-year survival	+	5	Benchmarking	Indicators were established, validated, and monitored. 40,000 patients have been included in the database. Results were reported periodically and submitted to realize auditing on an annual basis.
	2-year survival	+			
	5-year survival	+			
Siracusaet al., (2014) [37]	Median FEV1	0^a^	13	Plan-do-check-act (PDCA) The Chronic Care Model	Several improvement interventions implemented between 2001 and 2007 with focus on patient and family engagement in CF care, improve access and use of data, individualized scheduling, improving vaccination rates, infection control aiway clearance, standardization of care processes, and forming and QI team.
	Median body mass index (BMI)	0^a^			
Peterson et al., (2015) [26]	Systolic blood pressure	0^a^	13	Plan-do-check-act (PDCA) Collaborative Care Model	The effect of 23 diabetes teams joining a quality collaborative on patient outcomes.
	HbA1c	0^a^			
	LDL	0^a^			
Han et al. (2016) [31]	Hospitalization with ambulatory care-sensitive conditions	+	7	No clear QI method	Using clinical registry data to identify patients who should receive reminders for preventive/follow-up care and send reminders to those patients. Generate a list of patients by condition to use for quality improvement.
	ED visits	+			
Lail et al., (2017) [32]	Disease remission	0^a^	13	The Chronic Care Model	Eighteen condition teams implemented interventions varying from: establishing pre-visit planning (PVP), identifying the target populations, selecting and measuring outcomes and supporting processes, building and implementing care coordination, and assessing and addressing self-management support. The teams were free to choose the interventions that they thought would work best.
	Disease control	0^a^			
	Quality of life	0^a^			
	Symptom management	0^a^			

^1^+ means that the result was statistically significant at a *p*-value of 0.05

^2^0 means that there was no significant improvement in outcomes

^3^0^a^ means that there was improvement, but significance was not tested or reported

### Quality of the studies

#### Rigor of quality improvement interventions

The overall quality of included articles was moderate (see Tables 3 and 4). On the 16 domains of the QI-MQCS four articles achieved a score of 13, which is the highest score among included studies [24, 26, 32, 37]. These articles are therefore considered to be of good quality. Four articles were ranked as moderate quality with a score of 12 [35, 39, 42, 44]. Five articles scored poorly on the QI-MQCS with a score ≤ 7, which is ranked as low quality [31, 33, 34, 38, 41].

**Table 3:** Scoring of the RAND QI-MQCS

	Dziuban et al., (1994) [39]	Adams et al. (1998) [43]	Halpin et al. (2004) [40]	Moller et al., (2005) [38]	Thomas et al., (2007) [24]	Carlhed et al., (2009) [42]	Peterson et al., (2008) [27]	Jakobsen et al., (2009) [33]	MacLean et al., (2009) [25]	Kraynack & MacBride (2009) [35]
1. Organizational motivation	Y	Y	Y	N	Y	Y	N	N	N	N
2. Intervention rationale	Y	Y	Y	Y	Y	Y	Y	N	Y	Y
3 Intervention description	Y	N	Y	Y	Y	Y	Y	N	Y	Y
4. Organizational characteristics	Y	Y	Y	N	N	Y	N	N	Y	Y
5. Implementation	Y	N	Y	Y	Y	Y	Y	N	Y	Y
6. Study design	Y	Y	N	N	Y	Y	Y	Y	Y	Y
7. Comparator	Y	N	Y	N	Y	Y	Y	N	Y	Y
8. Data source	Y	Y	Y	Y	Y	Y	Y	Y	Y	Y
9. Timing	Y	Y	Y	N	Y	Y	Y	N	Y	Y
10. Adherence and fidelity	N	N	Y	N	N	N	N	N	N	Y
11. Health outcomes	Y	Y	Y	Y	Y	Y	Y	Y	Y	Y
12. Organizational readiness	Y	N	Y	N	Y	N	N	N	N	Y
13. Penetration and reach	N	N	N	N	N	Y	Y	Y	Y	N
14. Sustainability	N	Y	N	Y	Y	N	N	N	N	N
15. Spread	N	Y	N	Y	Y	N	N	N	N	Y
16. Limitations	Y	Y	N	N	Y	Y	Y	Y	N	N
*Total score (Y)*	*12*	*10*	*11*	*7*	*13*	*12*	*10*	*5*	*10*	*12*

**Table 4:** Scoring of the RAND QI-MQCS

	Bricker et al. (2010) [28]	Beaulieau et al. (2010) [41]	Bauer et al. (2011) [44]	Stern et al., (2011) [36]	Jakobsen et al., (2013) [34]	Siracusa et al., (2014) [37]	Peterson et al., (2015) [26]	Han et al., (2016) [31]	Lail et al., (2017) [32]	Toh et al., (2009) [30]	Baty et al., (2010) [29]
1. Organizational motivation	N	Y	Y	N	N	Y	N	N	Y	Y	Y
2. Intervention rationale	Y	Y	Y	Y	Y	Y	Y	Y	Y	Y	Y
3 Intervention description	N	Y	Y	Y	Y	Y	Y	Y	Y	Y	Y
4. Organizational characteristics	N	N	Y	Y	N	Y	Y	N	N	N	Y
5. Implementation	Y	Y	Y	Y	N	Y	Y	N	Y	Y	Y
6. Study design	Y	N	Y	N	N	N	Y	N	N	N	N
7. Comparator	Y	N	Y	Y	N	Y	Y	Y	N	Y	Y
8. Data source	Y	Y	Y	Y	Y	Y	Y	Y	Y	Y	Y
9. Timing	Y	N	Y	Y	N	Y	Y	Y	Y	Y	N
10. Adherence and fidelity	N	N	N	N	N	Y	Y	N	Y	N	N
11. Health outcomes	Y	Y	Y	Y	Y	Y	Y	Y	Y	Y	Y
12. Organizational readiness	N	Y	Y	N	N	Y	N	N	Y	Y	Y
13. Penetration and reach	Y	Y	N	N	Y	Y	N	N	Y	N	N
14. Sustainability	Y	N	N	Y	N	Y	Y	N	Y	N	N
15. Spread	N	N	N	Y	N	N	Y	N	Y	Y	N
16. Limitations	N	N	Y	N	N	N	Y	Y	Y	Y	Y
*Total score (Y)*	*9*	*7*	*12*	*10*	*5*	*13*	*13*	*7*	*13*	*11*	*10*

#### Rigor of data collection and analysis

The overall results of the quality assessment on data collection and analyses are displayed in the online supplementary content (Additional file 1: Appendix 5). Four studies have applied generalized linear mixed models for the analysis of change in outcomes [25, 27, 36, 42]. One study used a generalized estimating equation model with repeated measurements [24]. Inferential statistics have also been used in the form of survival analyses, logistic regression and chi-square analyses [29, 31, 33, 39, 44]. The remaining studies made use of descriptive statistical analyses only [26, 30, 32, 38, 43]. In order to monitor change, run charts have been applied in five studies [28, 35, 37, 40, 41].

On the additional item criteria, two studies have applied methods to account for missing values in their data, while also conducting a power analysis [25, 27].

### Methods used to achieve improvements

We identified six methods to achieve QI: benchmarking [33, 34, 38–41], a collaborative care model [26, 28, 42, 44], Plan-Do-Check-Act [36, 37], the Chronic Care Model [25, 32, 37], Learning and Leadership Collaborative [35] and IT driven interventions [24, 27, 29, 30, 41]. There were some studies where no clear QI method was used [29, 31, 43]. We will discuss these methods in the following paragraphs.

### Benchmarking

Benchmarking has been applied in several of the included studies [33, 34, 38, 39, 41]. Data was mostly compared among different hospitals [33, 34, 38]. Annual publication of data in the form of reports has most commonly been applied to report on results [33, 34, 41].

One study complemented their national report with an additional disease specific report with supplementary measures [33]. Another method of benchmarking was a discussion of the results at a (monthly or annual) meeting. During the annual meeting, results from reports were discussed and further evaluated [38]. Also, short-term feedback cycles with monthly publication of reports were applied [39]. The use of a strong data-driven system in combination with audits was characteristic of initiatives that applied benchmarking in order to improve outcomes as well as a model to change practice [33, 34, 39, 40].

### Collaborative care model

Three studies applied the Breakthrough Collaborative Model (BCM) to structure the goal of improving outcomes [26, 28, 42]. One study applied a Web-based disease registry to track patients with symptoms of depression to support treatment management in primary care [44]. In addition, evidence-based depression management training was provided to primary care providers. Moreover, in all sites, most patients experienced meaningful improvement in depression.

The BCM was used to design a cycle of structured discussion sessions during which outcomes were analyzed, presented and variation in work processes were discussed [26, 28]. The model was furthermore used as a guide to facilitate improvement efforts and insights into data [26, 42].

### Plan-do-check-act

In two studies Plan-Do-Check-Act (PDCA) cycles were used to improve outcomes and/or processes [26, 36, 37]. Yet, the cycle was presented as a supporting tool to other methods, either for the application of the BCM [26] or for benchmarking [36]. For the latter it was applied as a method to prepare for national benchmarking by organizing three PDCA cycles before data was shared [36]. The method was applied by organizing multidisciplinary meetings, where outcomes were discussed and improvement initiatives were identified [36]. Three cycles were organized in order to prepare public benchmarking after the third cycle [36].

The other study, which primarily used the methods outlined for the BCM, used the PDCA to structure and evaluate the learning sessions [26]. However, it was not the primary method for improving outcomes. In another study PDCA was used to continually evaluate local cystic fibrosis care practices, and they were able to improve pulmonary function and nutritional outcomes [37].

### The chronic care model

Three studies applied the Chronic Care Model (CCM) [25, 32, 37]. One study that applied the CCM used supporting techniques such as: audit and feedback, electronic registry, clinician reminders, patient reminders, and abbreviated patient education. It is, thus, rather a framework offering practical tools [25]. They did not find expected improvements in outcomes. Here, authors suggested that another, more collaborative approach would be needed to improve outcomes of chronic diseases [25]. The second study applied the CCM in children with various chronic conditions, in combination with PDCA cycles, failure mode and effect analysis and Pareto charts of failures [32]. This study resulted in improvement of respective outcomes [32]. The third study applied the CCM to ensure that all aspects of cystic fibrosis management were covered, and combined this with the PDCA to continually evaluate the processes of best practices in cystic fibrosis care. They did not evaluate the effectiveness of applying the CCM.

### Learning and leadership collaborative

The Learning and Leadership Collaborative (LLC) was applied in one study [35]. Commitment of a team to participate in a QI program, developing a sense of common responsibility as an organization for the improvement, measuring outcomes and processes and patient involvement were defined as key ingredients for QI. LLC has been used for training staff towards structured discussions on outcomes and/or processes and the introduction of a patient registry [35]. Data was registered and analyzed at one particular hospital, but presented to all participating hospitals. Participation in the LLC has led to the initiation of an improvement initiative at the hospital where the data were registered and analyzed.

### IT application as feedback tool

Five studies made used of (self-developed) IT applications, to empower patients and/or physicians to manage patients with greater care. The studies aimed at linking administrative and key clinical data and made use of reminder functions [24, 27, 30]. One study concluded their patients received better overall coordination of care [30]. Another two studies reported significant improvements in the percentage of type 2 diabetic patients and at-risk populations utilizing diabetes registries achieving recommended values for SBP, LDL, and HbA1C [27]. In one study, data were in addition displayed in operating room theatre, surgical office suites and nursing units [41]. Another study reported improved adherence to diabetes care processes in a continuity clinic due to the registry-generated audit, feedback, and patient reminders [24].

### Facilitators for quality improvements

A noticeable facilitator leading to QI was frequent reporting and feedback either annually or even monthly [28, 33, 34, 38–41]. The use of a database with high quality data, audits and reports as well as a strong stakeholder involvement were also found to be important factors contributing to successful QI [33, 34]. Structured registry data and an improvement intervention that can be linked to outcomes led to improvement in respective outcome measures [42]. In addition, other factors mentioned that would be needed for successful QI in one or more of the included studies are (1) patient involvement, communication, and standardization; (2) attitude and enthusiastic commitment from physicians, clinical managers and central administration and (3) appreciation concerning the importance of measurements [28, 35, 40, 41]. Moreover, improvement in outcomes appeared to be successful if supported by a proven QI approach [42]. Inconsistencies were found regarding the importance of involving an expert in the field of QI. On the one hand, involvement of a QI expert was considered positive for the start of an improvement agenda as it contributed to a more rapid implementation of improvement initiatives [42]. On the other hand, involving no additional expert or formal team was not experienced as a contributing factor to the success of outcome improvement [26]. This was only possible because a structured data registry was already present [26].

### Catalyst to improve outcomes over time

Outcomes can be improved over time through systematic use of outcome registries and facilitators. Outcome data and its interpretation help to achieve improvements in outcomes over time even faster compared to studies that did not use outcome data [34]. It was stated that outcomes were not only used to identify possible improvement interventions but also to monitor and secure improvements in the long run [34].

A computerized system was presented as a success factor to accelerate data from clinical registries to change outcomes and/or processes [26–29, 31–36, 42, 45]. Such a computerized system ensured valid and timely results [33]. Moreover, it allows for real-time feedback, which, in turn, leads to faster identification of improvement areas [28, 29, 31, 42].

Further use of outcome data for outcome improvement included the development of checklists, improved use of diagnostic standards, creation of data transparency, guidelines, improved patient recall, patient empowerment and leadership towards improvement [28, 29, 31, 36].

## Discussion

Eight out of the 21 included studies reported statistically significant improvements in outcomes including long-term survival, mortality, readmission rate, bleeding complications, systolic blood pressure, HbA1C, LDL, exercise habits (FEV1), depression improved in the acute phase (PHQ-9 score) and hospitalization with ambulatory care-sensitive conditions resulting from the implementation of QI initiatives. Out of these eight studies, the Chronic Care Model, IT application as feedback, benchmarking and the Collaborative Care Model were used as QI methods. A diverse set of clinical outcomes were collected and no patient-reported outcome measures (PROMs) were applied in any of the studies. Yet, only one study that reported statistically significant improvements in outcomes was of good quality. The improvement interventions were diverse, ranging from the implementation of guidelines, development of physician/patient alerts, improved teamwork, patient engagement methods through IT applications and the development of a supportive decision system. Many improvement interventions were combined in order to build a multifaceted approach to QI [24, 27, 28, 32, 37, 42, 44]. Facilitators for realizing QI include a high quality database, the use of pre-defined outcome measures, audits, frequent reporting and feedback, patient involvement, improved communication and standardization. Systematic approaches were used for structuring the improvement cycle. In order to use data from clinical registries as a catalyst to change outcomes, this review suggests that having a strong computerized system is supportive in aiding frontline clinical process management and improvement work.

A facilitator identified in this review was the organization of discussions for mapping and selecting best practices. It was further shown that a sound data management has a catalyzing effect. This data can be aggregated in annual reports, while it can also be used to compare with peers and/or perform nationwide comparisons. Also, a registry can facilitate access to real-time outcome and process data which can engage the team in realizing active improvements. Other registry programs such as the Get With The Guidelines-Stroke study, a large registry and performance improvement program for hospitalized patients with stroke and transient ischemic attack, also use annual reports for benchmark and feedback purposes [46].

Other systematic reviews concluded that audit and feedback can lead to small but important improvements in professional practice and healthcare outcomes [47]. They furthermore concluded that the effectiveness of audit and feedback depends on how the feedback was provided as well as on baseline performance. In addition, comparing this review to ours, there was one paper we have both included [24]. However, the objectives are very different, which can explain that there was not more overlap in included studies.

In addition, barriers and success factors to the effectiveness of feedback have been identified [48]. However, the authors were not able to draw sound conclusions on the effect of feedback on the quality of care and its potential to improve outcomes. Another review concerning renal registry data reflected on the potential of registry data and help advancing the nephrology care delivery [49].

None of the reviews studied the effect of QI efforts, besides from audit and feedback, on the quality of care and outcomes. This is the first study for which the literature was searched in detail in order to identify barriers and facilitators supporting QI interventions based on information from clinical registries.

The use of clinical registries can be seen as an important tool in order to systematically measure clinical outcomes and to achieve the goals of value-based health care. This is not only in line with our conclusions, but also acknowledged by others [1, 50, 51]. Other data sources can also be valuable for QI efforts, such as data from randomized controlled trials. However, this review aimed at including studies where structural data was collected through the use of a clinical registry.

In order to improve value, measuring both one or more outcomes and costs is essential [17]. Working with international registries makes it possible to make global comparisons, for example identifying practice variations and therefore improving quality of care for the whole patient group [52].

### Implications

We did not observe many efforts to incorporate PROMs. It is, however, generally considered important to measure the impact on health related quality of life (HRQoL) in the evaluation of the effect of QI initiatives [53]. The studies included for this review did not reflect on why they did not use PROMs and what would be the added value if they did. Even so, one study does report however the start of measuring quality of life in patients with cystic fibrosis [36]. The authors report this will lead to more insights into the complexity of QI efforts and personal patient gains in the experienced quality of life. It will also enable reporting on to what extent value was created from the patient’s perspective. Future QI efforts very likely combine QI with benchmarking incorporating quality of life outcomes.

None of the included studies reported costs, causing our study to be unable to evaluate the true impact on value. Incorporating costs will enable to identify cost drivers and comparing improvement interventions as proposed by the value-based healthcare principles [50]. A recent study showed that surgery for the oldest patients with colorectal cancer did not lead to increased hospital costs [51]. However, this study did identify variation in cost driver distribution. Patients under 85 years old had lower costs looking at the ward, operation and intensive care unit. Therefore, identifying costs and its main drivers will enable to develop improvement programs for specific sub-groups. This might be a powerful tool to reduce e.g. complications and thus hospital costs. Value-based health care could be the overarching concept guiding improvement initiatives, combined with well-defined methods. However, the field lacks a clear guide on implementation examples. Studies reflecting on impact, outcomes and costs are needed. Finally, the standardization of outcome measures is key, although they should be defined for a specific patient population. Transparent measurement of outcomes and costs has the potential to improving the value of care for all patients. Both providers, patients and payers can benefit from this collective common goal of transparency.

### Limitations

This review has some inherent limitations. Firstly, due to the very heterogeneous types of QI programs and their respective patient groups, it is difficult to generalize the results achieved in the included studies. Moreover, our inclusion criteria for QI programs may be to some extent arbitrary, which could possible lead to a bias in inclusion or exclusion of studies.

Also, the context in which the clinical registry is organized can impact outcomes. Moreover, important differences were observed in e.g. whether the registry was linked to reimbursement or public reporting versus primarily initiated for scientific or QI purposes or whether it was a voluntary or mandatory registry.

Secondly, the studies included in this review mainly focused on experiences in non-communicable diseases and thus often chronic patient groups. However, our aim was not to exclude communicable diseases from the study but we did not identify any studies in our literature search. This could indicate that chronic patient groups benefitted most from the realization of registries and respective QI interventions. As a result, improvement projects concerning other (non-chronic) patient groups have not been included in this review. Thirdly, due to publication bias, studies reporting no effect will be very likely not published and therefore missed out. Finally, two studies randomized practices [25, 27]. One study randomly allocated 19 volunteering hospitals to 1 of 2 intervention groups, where the intervention differed both in design and intensity [42]. In the other studies it should be noted that complete randomization was not possible, since the intervention hospitals involved were e.g. volunteering. Therefore, these hospitals might differ in their willingness to improve, causing potential selection bias.

## Conclusion

The results from this evaluation of studies which use outcome measures from clinical registry data to implement and monitor QI initiatives may help policy makers, managers and clinicians to understand the effectiveness, practicality and challenges of implementing QI interventions. An active and systematic approach is needed to improve outcomes. Continuous feedback from the data linked to clinical practice is crucial. Our review indicates that successful QI and consequently improved outcomes, is dependent on an active approach and organizational readiness.

There are many QI methods, and the majority of improvement interventions contain a combination of several methods. Clinical registries can be seen as supportive instruments in the process of improving quality of care. However, a clinical registry can only be successful in realizing QI efforts when there is commitment and leadership at both the physician and manager level, as well as a benchmarking facility, a well-integrated computerized system, and a collective aim to identify best practices.

## Additional files

Additional file 1: Appendix 1. Caption: Search string PubMed, Embase and Cochrane. Appendix 2. Caption: Eligibility Form Data collection form for intervention reviews: RCTs and non-RCTs. Appendix 3. Caption: Quality Improvement Minimum Quality Criteria Set (QI-MQCS) items. From: Hempel, Susanne, Paul G. Shekelle, Jodi L. Liu, Margie Sherwood Danz, Robbie Foy, Yee-Wei Lim, Aneesa Motala, and Lisa V. Rubenstein. Development of the Quality Improvement Minimum Quality Criteria Set (QI-MQCS): a tool for critical appraisal of quality improvement intervention publications. BMJ quality & safety (2015): bmjqs-2014. Appendix 4. Caption: Detailed Summary of Included Studies. Appendix 5. Caption: Scoring of the Downs & Black criteria, SQUIRE guidelines and additional self-developed tool. Notes: ^a^ From the Downs & Black questionnaire, question 2 and 18 have been used (Downs & Black 1998). ^b^ From the SQUIRE guidelines, question 10c,11a and 11b have been used (Ogrinc et al., 2008). (DOCX 101 kb)

## Abbreviations

BCM: the Breakthrough Collaborative Model
CCM: Chronic Care Model
FEV1: Forced expiratory volume in one second
HbA1C: Glycated Hemoglobin
HRQoL: Health related quality of life
LDL: Low-density lipoprotein
LLC: Learning and Leadership Collaborative
PDCA: Plan-Do-Check-Act
PROMs: Patient-reported outcome measures
QI: Quality Improvement
QI-MQSC: Quality Improvement Minimum Quality Criteria Set
SQUIRE: Standards for Quality Improvement Reporting Excellence
