# Supplementary material for: Health outcomes measurement and organizational readiness support quality improvement: a systematic review
Source: BMC Health Serv Res. 2018 Dec 29;18:1005. doi: 10.1186/s12913-018-3828-9 (PMC6311059; doi:10.1186/s12913-018-3828-9)
Supplement: Supplementary file 1 — Appendix 1. Caption: Search string PubMed, Embase and Cochrane. Appendix 2. Caption: Eligibility Form Data collection form for intervention reviews: RCTs and non-RCTs. Appendix 3. Caption: Quality Improvement Minimum Quality Criteria Set (QI-MQCS) items. From: Hempel, Susanne, Paul G. Shekelle, Jodi L. Liu, Margie Sherwood Danz, Robbie Foy, Yee-Wei Lim, Aneesa Motala, and Lisa V. Rubenstein. Development of the Quality Improvement Minimum Quality Criteria Set (QI-MQCS): a tool for critical appraisal of quality improvement intervention publications. BMJ quality & safety (2015): bmjqs-2014. Appendix 4. Caption: Detailed Summary of Included Studies. Appendix 5. Caption: Scoring of the Downs & Black criteria, SQUIRE guidelines and additional self-developed tool. Notes: a From the Downs & Black questionnaire, question 2 and 18 have been used (Downs & Black 1998). b From the SQUIRE guidelines, question 10c,11a and 11b have been used (Ogrinc et al., 2008). (DOCX 101 kb) [file 12913_2018_3828_MOESM1_ESM.docx]

**Additional file 1**

**Appendix 1.** Search string PubMed, Embase and Cochrane.

**Appendix 2**. Eligibility Form Data collection form for intervention reviews: RCTs and non-RCTs.

**Appendix 3**. Quality Improvement Minimum Quality Criteria Set (QI-MQCS) items.

**Appendix 4.** Detailed Summary of Included Studies.

**Appendix 5.** Scoring of the Downs & Black criteria, SQUIRE guidelines and additional self-developed tool.

This supplementary material has been provided by the authors to give readers additional information about their work.

**Appendix 1**. Search string for PubMed, Embase and Cochrane.

**PubMed**

("Mortality"[Mesh] OR "Patient Outcome Assessment"[Mesh] OR "Treatment Outcome"[Mesh] OR mortality[tiab] OR patient outcome*[tiab] OR patient reported outcome*[tiab] OR patient relevant outcome*[tiab] OR treatment outcome*[tiab] OR clinical outcome*[tiab] OR "outcome of care"[tiab] OR "outcomes of care"[tiab]) AND ("quality improvement registry" [tiab] OR "quality improvement registries" [tiab] OR "quality improvement register" [tiab] OR "quality registry" [tiab] OR "quality registries" [tiab] OR "quality register" [tiab] OR "device registry" [tiab] OR "device registries”[tiab] OR "device register" [tiab] OR "pregnancy registry" [tiab] OR "pregnancy registries" [tiab] OR "pregnancy register" [tiab] OR "disease registry"[tiab] OR "disease registries"[tiab] OR "disease register"[tiab] OR "medical registry"[tiab] OR "medical registries"[tiab] OR "medical register"[tiab] OR “patient registry”[tiab] OR ”patient registries" [tiab] OR "patient register" [tiab] OR "clinical registry"[tiab] OR "clinical registries"[tiab] OR "clinical register"[tiab] OR ”clinical data registry"[tiab] OR "clinical data registries”[tiab] OR ”clinical data register”[tiab] OR "outcome registry"[tiab] OR "outcome registries"[tiab] OR "outcome register"[tiab] OR "outcomes registry"[tiab] OR "outcomes registries"[tiab] OR "outcomes register"[tiab] OR "cardiac registry"[tiab] OR "cardiac registries"[tiab] OR "cardiac register"[tiab] OR "cardiovascular registry"[tiab] OR "cardiovascular registries"[tiab] OR "cardiovascular register"[tiab] OR "stroke registry"[tiab] OR "stroke registries"[tiab] OR "stroke register"[tiab] OR "cancer registry"[tiab] OR "cancer registries"[tiab] OR "cancer register"[tiab] OR "diabetes registry"[tiab] OR "diabetes registries"[tiab] OR "diabetes register"[tiab] OR "chronic disease registry" [tiab] OR "chronic disease registries"[tiab] OR "chronic disease register"[tiab] OR "rare disease registry"[tiab] OR "rare disease registries"[tiab] OR "rare disease register" [tiab] OR "paediatric registry"[tiab] OR "pediactric registries"[tiab] OR "paediatric register"[tiab] OR "psychiatric registry"[tiab] OR "psychiatric registries"[tiab] OR "psychiatric register"[tiab] OR “respiratory tract registry"[tiab] OR "respiratory tract registries"[tiab] OR "respiratory tract register"[tiab] OR "anesthesia registry"[tiab] OR "anesthesia registries"[tiab] OR "anesthesia register"[tiab] OR "intensive care registry"[tiab] OR "intensive care registries"[tiab] OR "intensive care register"[tiab] OR "circulation registry"[tiab] OR "circulation registries"[tiab] OR "circulation register"[tiab] OR "musculoskeletal registry"[tiab] OR "musculoskeletal registries"[tiab] OR "musculoskeletal register"[tiab] OR "orthopaedic registry"[tiab] OR "orthopaedic registries"[tiab] OR "orthopaedic register"[tiab] OR "rehabilitation registry"[tiab] OR "rehabilitation registries"[tiab] OR "rehabilitation register"[tiab] OR "rheumatology registry"[tiab] OR "rheumatology registries”[tiab] OR "rheumatology register"[tiab] OR "oral health registry"[tiab] OR "oral health registries"[tiab] OR "oral health register"[tiab] OR "eye disorder registry"[tiab] OR "eye disorder registries"[tiab] OR "eye disorder register"[tiab] OR "endocrinology registry"[tiab] OR "endocrinology registries"[tiab] OR " endocrinology register"[tiab] OR "infectious disease registry"[tiab] OR "infectious disease registries"[tiab] OR "infectious disease register"[tiab] OR "gastroenterology registry"[tiab] OR "gastroenterology registries"[tiab] OR "gastroenterology register"[tiab] OR "neurology registry"[tiab] OR "neurology registries"[tiab] OR "neurology register"[tiab] OR "obstetric registry"[tiab] OR "obstetric registries"[tiab] OR "obstetric register"[tiab] OR "gynaecology registry"[tiab] OR "gynaecology registries"[tiab] OR "gynaecology register"[tiab] OR "surgery registry"[tiab] OR "surgery registries"[tiab] OR "surgery register"[tiab] OR "cardiology registry"[tiab] OR "cardiology registries"[tiab] OR "cardiology register"[tiab] OR "infection control registry" [tiab] OR "infection control registries" [tiab] OR "infection control register”[tiab] OR “screening registry”[tiab] OR "screening registries" [tiab] OR "screening register" [tiab] OR "transplantation registry" [tiab] OR "transplantation registries" [tiab] OR "transplantation register”[tiab] OR "trauma registry" [tiab] OR "trauma registries" [tiab] OR "trauma register" [tiab])

**Embase**

('mortality'/exp OR 'treatment outcome'/de OR mortality:ab,ti OR ((patient OR 'patient reported' OR 'patient relevant' OR 'clinical' OR ‘treatment') NEXT/1 outcome*):ab,ti) OR 'outcome of care'/de AND ('quality improvement registry' OR 'quality improvement registries' OR 'quality improvement register' OR 'device registry' OR 'device registries' OR 'device register' OR 'pregnancy registry' OR 'pregnancy registries' OR 'pregnancy register' OR 'disease registry' OR 'disease registries' OR 'disease register' OR 'patient registry' OR 'patient registries' OR 'patient register' OR 'medical registry' OR 'medical registries' OR 'medical register' OR 'clinical registry' OR 'clinical registries' OR 'clinical register' OR 'clinical data registry' OR 'clinical data registries' OR 'clinical data register' OR 'outcome registry' OR 'outcome registries' OR 'outcome register' OR 'outcomes registry' OR 'outcomes registries' OR 'outcomes register' OR 'cardiac registry' OR 'cardiac registries' OR 'cardiac register' OR 'cardiovascular registry' OR 'cardiovascular registries' OR 'cardiovascular register' OR 'stroke registry' OR 'stroke registries' OR 'stroke register' OR 'cancer registry' OR 'cancer registries' OR 'cancer register' OR 'diabetes registry' OR 'diabetes registries' OR 'diabetes register' OR 'chronic disease registry' OR 'chronic disease registries' OR 'chronic disease register' OR 'rare disease registry' OR 'rare disease registries' OR 'rare disease register' OR 'paediatric registry' OR 'pediactric registries' OR 'paediatric register' OR 'psychiatric registry' OR 'psychiatric registries' OR 'psychiatric register' OR 'respiratory tract registry' OR 'respiratory tract registries' OR 'respiratory tract register' OR 'anesthesia registry' OR 'anesthesia registries' OR 'anesthesia register' OR 'intensive care registry' OR 'intensive care registries' OR 'intensive care register' OR 'circulation registry' OR 'circulation registries' OR 'circulation register' OR 'musculoskeletal registry' OR 'musculoskeletal registries' OR 'musculoskeletal register' OR 'orthopaedic registry' OR 'orthopaedic registries' OR 'orthopaedic register' OR 'rehabilitation registry' OR 'rehabilitation registries' OR 'rehabilitation register' OR 'rheumatology registry' OR 'rheumatology registries' OR 'rheumatology register' OR 'oral health registry' OR 'oral health registries' OR 'oral health register' OR 'eye disorder registry' OR 'eye disorder registries' OR 'eye disorder register' OR 'endocrinology registry' OR 'endocrinology registries' OR 'endocrinology register' OR 'infectious disease registry' OR 'infectious disease registries' OR 'infectious disease register' OR 'gastroenterology registry' OR 'gastroenterology registries' OR 'gastroenterology register' OR 'neurology registry' OR 'neurology registries' OR 'neurology register' OR 'obstetric registry' OR 'obstetric registries' OR 'obstetric register' OR 'gynaecology registry' OR 'gynaecology registries' OR 'gynaecology register' OR 'surgery registry' OR 'surgery registries' OR 'surgery register' OR 'cardiology registry' OR 'cardiology registries' OR 'cardiology register' OR 'infection control registry' OR 'infection control registries' OR 'infection control register' OR 'screening registry' OR 'screening registries' OR 'screening register' OR 'transplantation registry' OR 'transplantation registries' OR 'transplantation register' OR 'trauma registry' OR 'trauma registries' OR 'trauma register'):ab,ti NOT 'conference abstract'/it

**Cochrane Library**

(mortality OR patient outcome* OR patient reported outcome* OR patient relevant outcome* OR treatment outcome*):ab,ti AND (“quality improvement registry” OR “quality improvement registries” OR “quality improvement register” OR “device registry” OR “device registries” OR “device register” OR “pregnancy registry” OR “pregnancy registries” OR “pregnancy register” OR “disease registry” OR “disease registries” OR “disease register” OR “patient registry” OR “patient registries” OR “patient register” OR “clinical registry” OR “clinical registries” OR “clinical register” OR “clinical data registry” OR “clinical data registries” OR “clinical data register” OR “outcome registry” OR “outcome registries” OR “outcome register” OR “outcomes registry” OR “outcomes registries” OR “outcomes register” OR “cardiac registry” OR “cardiac registries” OR “cardiac register” OR “cardiovascular registry” OR “cardiovascular registries” OR “cardiovascular register” OR “stroke registry” OR “stroke registries” OR “stroke register” OR “cancer registry” OR “cancer registries” OR “cancer register” OR “diabetes registry” OR “diabetes registries” OR “diabetes register” OR “chronic disease registry” OR “chronic disease registries” OR “chronic disease register” OR “rare disease registry” OR “rare disease registries” OR “rare disease register” OR “paediatric registry” OR “pediactric registries” OR “paediatric register” OR “psychiatric registry” OR “psychiatric registries” OR “psychiatric register” OR “respiratory tract registry” OR “respiratory tract registries” OR “respiratory tract register” OR “anesthesia registry” OR “anesthesia registries” OR “anesthesia register” OR “intensive care registry” OR “intensive care registries” OR “intensive care register” OR “circulation registry” OR “circulation registries” OR “circulation register” OR “musculoskeletal registry” OR “musculoskeletal registries” OR “musculoskeletal register” OR “orthopaedic registry” OR “orthopaedic registries” OR “orthopaedic register” OR “rehabilitation registry” OR “rehabilitation registries” OR “rehabilitation register” OR “rheumatology registry” OR “rheumatology registries” OR “rheumatology register” OR “oral health registry” OR “oral health registries” OR “oral health register” OR “eye disorder registry” OR “eye disorder registries” OR “eye disorder register” OR “endocrinology registry” OR “endocrinology registries” OR “endocrinology register” OR “infectious disease registry” OR “infectious disease registries” OR “infectious disease register” OR “gastroenterology registry” OR “gastroenterology registries” OR “gastroenterology register” OR “neurology registry” OR “neurology registries” OR “neurology register” OR “obstetric registry” OR “obstetric registries” OR “obstetric register” OR “gynaecology registry” OR “gynaecology registries” OR “gynaecology register” OR “surgery registry” OR “surgery registries” OR “surgery register” OR “cardiology registry” OR “cardiology registries” OR “cardiology register” OR “infection control registry” OR “infection control registries” OR “infection control register” OR “screening registry” OR “screening registries” OR “screening register” OR “transplantation registry” OR “transplantation registries” OR “transplantation register” OR “trauma registry” OR “trauma registries” OR “trauma

**Appendix 2.** Eligibility Form Data collection form for intervention reviews: RCTs and non-RCTs.


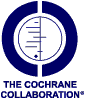


Data collection form for intervention reviews: RCTs and non-RCTs

Version 1, may 2016

This form can be used as a guide for developing your own data extraction form. Sections can be expanded and added, and irrelevant sections can be removed. It is difficult to design a single form that meets the needs of all reviews, so it is important to consider carefully the information you need to collect, and design your form accordingly. Information included on this form should be comprehensive, and may be used in the text of your review, 'Characteristics of included studies' table, risk of bias assessment, and statistical analysis.

Using this form, or an adaptation of it, will help you to meet [MECIR standards](http://www.editorial-unit.cochrane.org/mecir) for collecting and reporting information about studies for your review, and analysing their results (see MECIR standards C43 to C55; R41 to R45).

## Notes on using data extraction form:

- Be consistent in the order and style you use to describe the information for each report.
- Record any missing information as unclear or not described, to make it clear that the information was not found in the study report(s), not that you forgot to extract it.
- Include any instructions and decision rules on the data collection form, or in an accompanying document. It is important to practice using the form and give training to any other authors using the form.

| **Title of the article/article/report** |  |
| --- | --- |
| **Study ID** *(surname of first author and year first full report of study was published e.g. Smith 2001)* |  |
| **Report ID** of other reports of this study including errata or retractions |  |
| **Notes** | |

1. General Information

| **1.1 Date form completed** *(dd/mm/yyyy)* |  |
| --- | --- |
| **1.2 Name/ID of person extracting data** |  |
| **1.3 Reference citation** |  |
| **1.4 Study author contact details** |  |
| **1.5 Publication type and Journal** *(e.g. full report, abstract, letter)* |  |
| **Notes:** | |

2. Characteristics of included studies

2.1 Methods

|  | **Descriptions as stated in report/paper** | | **Location in text or source** *(pg & ¶/fig/table/other)* |
| --- | --- | --- | --- |
| **2.1.1 Aim of study** *(e.g. efficacy, equivalence, pragmatic)* |  | |  |
| **2.1.2 Design** *(e.g. parallel, crossover, non-RCT)* |  | |  |
| **2.1.3 Allocation of comparison** *(by individuals, cluster/ groups or body parts)* |  | |  |
| **2.1.4 Start date** |  | |  |
| **2.1.5 End date** |  | |  |
| **2.1.6 Duration of participation** *(from recruitment to last follow-up)* |  | |  |
| **2.1.7 Ethical approval needed/ obtained for study** | Yes No Unclear |  |  |
| **Notes:** | | | |

3.2 Participants

|  | Description  *Include comparative information for each intervention or comparison group if available* | | Location in text or source *(pg & ¶/fig/table/other)* |
| --- | --- | --- | --- |
| **3.2.1 Population description** *(from which study participants are drawn)* |  | |  |
| **3.2.2 Setting** *(including location and social context)* |  | |  |
| **3.2.3 Inclusion criteria** |  | |  |
| **3.2.4 Exclusion criteria** |  | |  |
| **3.2.5 Method of recruitment of participants** *(e.g. phone, mail, clinic patients)* |  | |  |
| **3.2.6 Informed consent obtained** | Yes No Unclear |  |  |
| **3.2.7 Total no. of pop. at start of study for NRCTs** |  | |  |
| **3.2.8 Clusters** *(if applicable, no., type, no. people per cluster)* |  | |  |
| **3.2.9 Baseline imbalances** |  | |  |
| **3.2.10 Age** |  | |  |
| **3.2.11 Sex** |  | |  |
| **3.2.12 Race/Ethnicity** |  | |  |
| **3.2.13 Severity of illness** |  | |  |
| **3.2.14 Co-morbidities** |  | |  |
| **3.2.15 Other relevant sociodemographics** |  | |  |
| **3.2.16 Subgroups measure** |  | |  |
| **3.2.17 Subgroups reported** |  | |  |
| **Notes:** | | | |

3.3 Intervention groups

*Copy and paste table for each intervention and comparison group*

**Intervention Group 1**

|  | Description as stated in report/paper | Location in text or source *(pg & ¶/fig/table/other)* |
| --- | --- | --- |
| **3.3.1 Group name** |  |  |
| **3.3.2 No. randomised to group** *(specify whether no. people or clusters)* |  |  |
| **3.3.3 Theoretical basis** *(include key references)* |  |  |
| **3.3.4 Description** *(include sufficient detail for replication, e.g. content, dose, components)* |  |  |
| **3.3.5 Duration of treatment period** |  |  |
| **3.3.6 Timing** *(e.g. frequency, duration of each episode)* |  |  |
| **3.3.7 Delivery** *(e.g. mechanism, medium, intensity, fidelity)* |  |  |
| **3.3.8 Providers** *(e.g. no., profession, training, ethnicity etc. if relevant)* |  |  |
| **3.3.9 Co-interventions** |  |  |
| **3.3.10 Economic information** *(i.e. intervention cost, changes in other costs as result of intervention)* |  |  |
| **3.3.11 Resource requirements** *(e.g. staff numbers, cold chain, equipment)* |  |  |
| **3.3.12 Integrity of delivery** |  |  |
| **Compliance** |  |  |
| **Notes:** | | |

3.4 Outcomes

*Copy and paste table for each outcome.*

**Outcome 1**

|  | Description as stated in report/paper | Location in text or source *(pg & ¶/fig/table/other)* |
| --- | --- | --- |
| **3.4.1 Outcome name** |  |  |
| **3.4.2 Time points measured** *(specify whether from start or end of intervention)* |  |  |
| **3.4.3 Time points reported** |  |  |
| **3.4.4 Outcome definition** *(with diagnostic criteria if relevant)* |  |  |
| **3.4.5 Person measuring/ reporting** |  |  |
| **3.4.6 Unit of measurement** *(if relevant)* |  |  |
| **3.4.7 Scales: upper and lower limits** *(indicate whether high or low score is good)* |  |  |
| Notes: | | |

3.5 Other

| **3.5.1 Study funding sources** *(including role of funders)* |  |  |
| --- | --- | --- |
| **3.5.2 Possible conflicts of interest** *(for study authors)* |  |  |
| **Notes:** | | |

5. Data and analysis

*Copy and paste the appropriate table for each outcome, including additional tables for each time point and subgroup as required.*

1. ***For RCT/CCT***

***Dichotomous outcome***

|  | Description as stated in report/paper | | | | | Location in text or source *(pg & ¶/fig/table/other)* |
| --- | --- | --- | --- | --- | --- | --- |
| **5.1a Comparison** |  | | | | |  |
| **5.2a Outcome** |  | | | | |  |
| **5.3a Subgroup** |  | | | | |  |
| **5.4a Time point** *(specify from start or end of intervention)* |  | | | | |  |
| **5.5a Results** | Intervention | | | Comparison | |  |
|  | No. with event | Total in group | | No. with event | Total in group |  |
|  |  |  | |  |  |  |
| **5.6a Any other results reported** *(e.g. odds ratio, risk difference, CI or P value)* |  | | | | |  |
| **5.7a No. missing participants** |  | | |  | |  |
| **5.8a Reasons missing** |  | | |  | |  |
| **5.9a No. participants moved from other group** |  | | |  | |  |
| **5.10a Reasons moved** |  | | |  | |  |
| **5.11a Unit of analysis** *(by individuals, cluster/groups or body parts)* |  | | | | |  |
| **5.12a Statistical methods used and appropriateness of these** *(e.g. adjustment for correlation)* |  | | | | |  |
| **5.13a Reanalysis required?** *(specify, e.g. correlation adjustment)* | Yes No Unclear | |  | | |  |
| **5.14a Reanalysis possible?** | Yes No Unclear | |  | | |  |
| **5.15a Reanalysed results** |  | | | | |  |
| Notes: | | | | | | |

1. ***For RCT/CCT***

***Continuous outcome***

|  | | Description as stated in report/paper | | | | | Location in text or source *(pg & ¶/fig/table/other)* | |
| --- | --- | --- | --- | --- | --- | --- | --- | --- |
| **5.1b Comparison** | |  | | | | |  | |
| **5.2b Outcome** | |  | | | | |  | |
| **5.3b Subgroup** | |  | | | | |  | |
| **5.4b Time point** *(specify from start or end of intervention)* | |  | | | | |  | |
| **5.5b Post-intervention or change from baseline?** | |  | | | | |  | |
| **5.6b Results** | Intervention | | | Comparison | | |  | |
|  | Mean | SD *(or other variance, specify)* | No. participants | Mean | SD *(or other variance, specify)* | No. participants |  |  |
|  |  |  |  |  |  |  |  |  |
| **5.7b Any other results reported** *(e.g. mean difference, CI, P value)* | |  | | | | |  | |
| **5.8b No. missing participants** | |  | |  | | |  |  |
| **5.9b Reasons missing** | |  | |  | | |  |  |
| **5.10b No. participants moved from other group** | |  | |  | | |  |  |
| **5.11b Reasons moved** | |  | |  | | |  |  |
| **5.12b Unit of analysis** *(individuals, cluster/ groups or body parts)* | |  | | | | |  | |
| **5.13b Statistical methods used and appropriateness of these** *(e.g. adjustment for correlation)* | |  | | | | |  | |
| **5.14b Reanalysis required?** *(specify)* | | Yes No Unclear | |  | | |  | |
| **5.15b Reanalysis possible?** | | Yes No Unclear | |  | | |  | |
| **5.16b Reanalysed results** | |  | | | | |  | |
| Notes: | | | | | | | | |

1. ***For RCT/CCT***

***Other outcome***

|  | Description as stated in report/paper | | | | | Location in text or source *(pg & ¶/fig/table/other)* |
| --- | --- | --- | --- | --- | --- | --- |
| **5.1c Comparison** |  | | | | |  |
| **5.2c Outcome** |  | | | | |  |
| **5.3c Subgroup** |  | | | | |  |
| **5.3c Time point** *(specify from start or end of intervention)* |  | | | | |  |
| **5.4c No. participant** | Intervention | | | Control | |  |
|  |  | | |  | |  |
| **5.5c Results** | Intervention result | SE (or other variance) | | Control result | SE (or other variance) |  |
|  |  |  | |  |  |  |
|  | Overall results | | | SE (or other variance) | |  |
|  |  | | |  | |  |
| **5.6c Any other results reported** |  | | | | |  |
| **5.7c No. missing participants** |  | | |  | |  |
| **5.8c Reasons missing** |  | | |  | |  |
| **5.9c No. participants moved from other group** |  | | |  | |  |
| **5.10c Reasons moved** |  | | |  | |  |
| **5.11c Unit of analysis** *(by individuals, cluster/groups or body parts)* |  | | | | |  |
| **5.12c Statistical methods used and appropriateness of these** |  | | | | |  |
| **5.13c Reanalysis required?** *(specify)* | Yes No Unclear | |  | | |  |
| **5.14c Reanalysis possible?** | Yes No Unclear | |  | | |  |
| **5.15c Reanalysed results** |  | | | | |  |
| **Notes:** | | | | | | |

1. ***For Controlled Before-and-After study (CBA)***

|  | Description as stated in report/paper | | | | | Location in text or source *(pg & ¶/fig/table/other)* |
| --- | --- | --- | --- | --- | --- | --- |
| **5.1d Comparison** |  | | | | |  |
| **5.2d Outcome** |  | | | | |  |
| **5.3d Subgroup** |  | | | | |  |
| **5.4d Time point** *(specify from start or end of intervention)* |  | | | | |  |
| **5.5d Post-intervention or change from baseline?** |  | | | | |  |
| **5.6d No. participants** | Intervention | | | Control | |  |
|  |  | | |  | |  |
| **5.7d Results** | Intervention result | SE *(or other variance, specify)* | | Control result | SE *(or other variance, specify)* |  |
|  |  |  | |  |  |  |
|  | Overall results | | | SE *(or other variance, specify)* | |  |
|  |  | | |  | |  |
| **5.8d Any other results reported** |  | | | | |  |
| **5.9d No. missing participants** |  | | |  | |  |
| **5.10d Reasons missing** |  | | |  | |  |
| **5.11d No. participants moved from other group** |  | | |  | |  |
| **5.12d Reasons moved** |  | | |  | |  |
| **5.13d Unit of analysis** *(individuals, cluster/ groups or body parts)* |  | | | | |  |
| **5.14d Statistical methods used and appropriateness of these** |  | | | | |  |
| **5.15d Reanalysis required?** *(specify)* | Yes No Unclear | |  | | |  |
| **5.16d Reanalysis possible?** | Yes No Unclear | |  | | |  |
| **5.17d Reanalysed results** |  | | | | |  |
| **Notes:** | | | | | | |

1. ***For Interrupted Time Series study (ITS)***

|  | Description as stated in report/paper | | | | | | Location in text or source *(pg & ¶/fig/table/other)* |
| --- | --- | --- | --- | --- | --- | --- | --- |
| **5.1e Comparison** |  | | | | | |  |
| **5.2e Outcome** |  | | | | | |  |
| **5.3e Subgroup** |  | | | | | |  |
| **5.4e Length of time points measured** *(e.g. days, months)* |  | | | | | |  |
| **5.5e Total period measured** |  | | | | | |  |
| **5.6e No. participants measured** |  | | | | | |  |
| **5.7e No. missing participants** |  | | | | | |  |
| **5.8e Reasons missing** |  | | | | | |  |
|  | Pre-intervention | | | | Post-intervention | |  |
| **5.9e No. time points measured** |  | | | |  | |  |
| **5.10e Mean value** *(with variance measure)* |  | | | |  | |  |
| **5.11e Any other results reported** |  | | | | | |  |
| **5.12e Unit of analysis** *(individuals or cluster/ groups)* |  | | | | | |  |
| **5.13e Statistical methods used and appropriateness of these** |  | | | | | |  |
| **5.14e Reanalysis required?** *(specify)* | Yes No Unclear | | |  | | |  |
| **5.15e Reanalysis possible?** | Yes No Unclear | | |  | | |  |
| **Individual time point results** |  | | | | | |  |
| **5.16e Read from figure?** | Yes No |  | | | | |  |
| **5.17e Reanalysed results** | Change in level | | SE | | Change in slope | SE |  |
|  |  | |  | |  |  |  |
| Notes: | | | | | | | |

6. Other information

|  | **Description as stated in report/paper** | **Location in text or source** *(pg & ¶/fig/table/other)* |
| --- | --- | --- |
| **6.1 Key conclusions of study authors** |  |  |
| **6.2 References to other relevant studies** |  |  |
| **6.3 Correspondence required for further study information** *(from whom, what and when)* |  | |
| **Notes:** | | |

7. Definitions

| Assumed risk estimate | An estimate of the risk of an event or average score without the intervention, used in Cochrane 'Summary of findings tables'. If a study provides useful estimates of the risk or average score of different subgroups of the population, or an estimate based on a representative observational study, you may wish to collect this information. |
| --- | --- |
| Bias | A systematic error or deviation in results or inferences from the truth. In studies of the effects of health care, the main types of bias arise from systematic differences in the groups that are compared (selection bias), the care that is provided, exposure to other factors apart from the intervention of interest (performance bias), withdrawals or exclusions of people entered into a study (attrition bias) or how outcomes are assessed (detection bias). Reviews of studies may also be particularly affected by reporting bias, where a biased subset of all the relevant data is available. |
| Change from baseline | A measure for a continuous outcome calculated as the difference between the baseline score and the post-intervention score. |
| Clusters | A group of participants who have been allocated to the same intervention arm together, as in a cluster-randomised trial, e.g. a whole family, town, school or patients in a clinic may be allocated to the same intervention rather than separately allocating each individual to different arms. |
| Co-morbidities | The presence of one or more diseases or conditions other than those of primary interest. In a study looking at treatment for one disease or condition, some of the individuals may have other diseases or conditions that could affect their outcomes. |
| Compliance | Participant behaviour that abides by the recommendations of a doctor, other health care provider or study investigator (also called adherence or concordance). |
| Contemporaneous data collection | When data are collected at the same point(s) in time or covering the same time period for each intervention arm in a study (that is, historical data are not used as a comparison). |
| Controlled Before and After Study (CBA) | A non-randomised study design where a control population of similar characteristics and performance as the intervention group is identified. Data are collected before and after the intervention in both the control and intervention groups |
| Exclusions | Participants who were excluded from the study or the analysis by the investigators. |
| Imputation | Assuming a value for a measure where the true value is not available (e.g. assuming last observation carried forward for missing participants). |
| Integrity of delivery | The degree to which the specified procedures or components of an intervention are delivered as originally planned. |
| Interrupted Time Series (ITS) | A research design that collects observations at multiple time points before and after an intervention (interruption). The design attempts to detect whether the intervention has had an effect significantly greater than the underlying trend. |
| Post-intervention | The value of an outcome measured at some time point following the beginning of the intervention (may be during or after the intervention period). |
| Power | In clinical trials, power is the probability that a trial will obtain a statistically significant result when the true intervention effect is a specified size. For a given size of effect, studies with more participants have greater power. Note that power should not be considered in the risk of bias assessment. |
| Providers | The person or people responsible for delivering an intervention and related care, who may or may not require specific qualifications (e.g. doctors, physiotherapists) or training. |
| Quasi-randomised controlled trial | A study in which the method of allocating people to intervention arms was not random, but was intended to produce similar groups when used to allocate participants. Quasi-random methods include: allocation by the person's date of birth, by the day of the week or month of the year, by a person's medical record number, or just allocating every alternate person. |
| Reanalysis | Additional analysis of a study's results by a review author (e.g. to introduce adjustment for correlation that was not done by the study authors). |
| Report ID | A unique ID code given to a publication or other report of a study by the review author (e.g. first author's name and year of publication). If a study has more than one report (e.g. multiple publications or additional unpublished data) a separate Report ID can be allocated to each to help review authors keep track of the source of extracted data. |
| Sociodemographics | Social and demographic information about a study or its participants, including economic and cultural information, location, age, gender, ethnicity, etc. |
| Study ID | A unique ID code given to an included or excluded study by the review author (e.g. first author's name and year of publication from the main report of the study). Although a study may have multiple reports or references, it should have one single Study ID to help review authors keep track of all the different sources of information for a study. |
| Theoretical basis | The use of a particular theory (such as theories of human behaviour change) to design the components and implementation of an intervention |
| Unit of allocation | The unit allocated to an intervention arm. In most studies individual participants will be allocated, but in others it may be individual body parts (e.g. different teeth or joints may be allocated separately) or clusters of multiple people. |
| Unit of analysis | The unit used to calculate N in an analysis, and for which the result is reported. This may be the number of individual people, or the number of body parts or clusters of people in the study. |
| Unit of measurement | The unit in which an outcome is measured, e.g. height may be measured in cm or inches; depression may be measured using points on a particular scale. |
| Validation | A process to test and establish that a particular measurement tool or scale is a good measure of that outcome. |
| Withdrawals | Participants who voluntarily withdrew from participation in a study before the completion of outcome measurement. |

**Sources:**

Cochrane Collaboration Glossary, 2010. Available from [www.cochrane.org/glossary](http://www.cochrane.org/glossary).

Higgins JPT, Green S (editors). Cochrane Handbook for Systematic Reviews of Interventions Version 5.1.0 [updated March 2011]. The Cochrane Collaboration, 2011. Available from [handbook.cochrane.org](http://handbook.cochrane.org).

Last JM (editor), A Dictionary of Epidemiology, 4^th^ Ed. New York: Oxford University Press, 2001.

Schünemann H, Brożek J, Oxman A, editors. GRADE handbook for grading quality of evidence and strength of recommendation. Version 3.2 [updated March 2009]. The GRADE Working Group, 2009.

**Appendix 3**. Quality Improvement Minimum Quality Criteria Set (QI-MQCS) items.

| **Items** | **Yes/No** |
| --- | --- |
| **1 Organisational motivation**  assesses whether the motivational context of the organisation in which the intervention was introduced was described; for example to convey whether a given quality problem—such as shortcomings in quality of care indicators—was being addressed. |  |
| **2 Intervention rationale**  assesses whether a rationale was given that suggests why the intervention  may produce improvements in the outcome (empirical evidence, theories or logic models).  **NOTE:**  - Quality improvement processes can be the intervention |  |
| **3 Intervention description (change in organizational or provider behaviour)**  requires a detailed description of the change in the structure or organisation of healthcare, including personnel involved. QI interventions are diverse and may address changes in care processes (eg, use of care managers) or strategies aiming to change provider behaviour (eg, electronicreminders), and the content (eg, avoiding catheterrelated blood stream infections), and the means to achieve the goal (eg, audit and feedback) are often intertwined. We restricted the definition to permanent structural or organisational changes, not temporary activities aiming to develop or introduce the change. |  |
| **4 Organisational characteristics**  assesses whether key demographics of the setting are described to provide  information that enables readers to assess the generalizability to their organisation.  **NOTE:**  - describing e.g. the number of patients out of XX county wide or number of clinics out of the XX clinics nationwide.  - Factors which are key/central for that particular population are described. |  |
| **5 Implementation**  addresses temporary activities used to introduce the permanent change, for example, staff education to introduce a new care protocol. The QI-MQCS focuses here on the introduction of the intervention into clinical practice, not its development. |  |
| **6 Study design**  assesses whether the evaluation design to determine whether the intervention was successful was identified. Acknowledging that different questions require different study designs, the quality emphasis is on outlining the evaluation approach, not on specific designs or features (eg, randomisation). |  |
| **7 Comparator**  assesses the control condition to which the intervention is compared, for example, routine care before the intervention was introduced. We added this  item, most prominently described in the Workgroup for Intervention Development and Evaluation Research (WIDER) criteria, in response to TEP discussions and empirical evidence. Given that healthcare contexts are continually evolving, it is important to know whether the comparison group comprised current ‘state-of-the-art’ or poor quality care |  |
| **8 Data source**  considers how data were obtained for the evaluation and whether the primary outcome was defined; conveying what exactly was measured  should avoid a ‘false implicit understanding’ of terms and definitions and is independent from the study design selected for the evaluation. |  |
| **9 Timing**  addresses the clarity of the timeline in relation to the evaluation of the intervention, for example, when a complex change was fully implemented and when evaluated, in order to determine the follow-up period. |  |
| **10 Adherence/fidelity**  addresses compliance with the intervention. QI interventions can be introduced with enthusiasm, but whether personnel actually adhere to them  (eg, a new assessment tool) in busy routine clinical practice is another matter. Readers need to be able to judge whether any intervention failure was attributable to the intervention itself, suboptimal translation in clinical  practice, or a combination of both. Any information on adherence (including the lack thereof) is acknowledged in assessing this domain. |  |
| **11 Health outcomes**  considers whether patient health outcomes are part of the evaluation. Although an intervention may result in changes in healthcare processes  (eg, tests ordered), they may not necessarily improve patient outcomes. The QI-MQCS acknowledges studies that assess this crucial patient-centered  question. |  |
| **12 Organisational readiness**  refers to the QI culture and resources present in the organisation, which helps to assess the transferability of results. |  |
| **13 penetration/reach**  assesses what proportion of eligible units participated. This domain requires a denominator; stating the number of participating sites without also reporting how many sites were initially approached or were eligible is not sufficient. |  |
| **14 sustainability**  addresses whether information on the sustainability of the intervention is available; including positive evidence (eg, an extended intervention  period) or acknowledgment that the intervention may be maintained only with additional resources. |  |
| **15 spread**  addresses the ability of the intervention to be spread to or replicated in other settings. The minimum quality standard is met if the potential or  unsuccessful attempts at spread or positive evidence of spread (eg, large-scale rollouts) are presented. |  |
| **16 limitations**  refers to disclosed limitations of the evaluation of the intervention. |  |

From: Hempel, Susanne, Paul G. Shekelle, Jodi L. Liu, Margie Sherwood Danz, Robbie Foy, Yee-Wei Lim, Aneesa Motala, and Lisa V. Rubenstein. Development of the Quality Improvement Minimum Quality Criteria Set (QI-MQCS): a tool for critical appraisal of quality improvement intervention publications. *BMJ quality & safety* (2015): bmjqs-2014.

**Appendix 4.** Detailed Summary of Included Studies.

| **Source** | **Design** | **Aim** | **Target group** | **Main results** | **Data source** |
| --- | --- | --- | --- | --- | --- |
| Peterson et al., (2015) | Case study design | To study how quality improvement collaboration (QIC) can impact clinical practice and outcomes for patients with diabetes mellitus. | Diabetes care delivery/ patients | QIC helped teams to improve patient outcomes compared to national average for systolic blood pressure, and low density lipoprotein levels. | National Diabetes Register (NDR) |
| Jakobsen et al., (2009) | Observational study | To study how to improve the quality of care through quality measures. | Lung cancer patients | - Overall 1- and 2-year survival improved: 69% and 50% in year 2000 to 77% and 60% in 2005, respectively (*p* = 0.001 and 0.004, respectively). - 30-day mortality after surgery decreased from 5.2% (2000) to 3.6% (2007). - Patients having surgery within 14 days from referral increased from 69% (2000) to 83% (2007). | Danish lung cancer registry |
| Peterson et al., (2008) | Group-RCT | To determine if the implementation of a multicomponent organizational intervention can impact diabetes care and outcomes in community primary care practices. | Diabetes | Diabetes process measures increased significantly more in intervention than in control practices: foot examinations 35.0% (*p* < 0.0.001); annual eye examinations 25.9% (*p* < 0.001); renal testing 28.5% (*p* < 0.001); A1C testing 8.1%(p < 0.001); blood pressure monitoring 3.5% (*P* = 0.05); and LDL testing 8.6% (*p* < 0.001). Mean A1C (adjusted for sex, age and comorbidity) decreased significantly in intervention practices (*p* < 0.02). At 12 months, intervention practices had significantly greater improvements in achieving recommended values for SBP, A1C, and LDL than control clinics (*p* = 0.002). | - |
| MacLean et al., (2009) | RCT | To evaluate the impact of a registry and decision support system on physiologic results & control and process of care. | Diabetes care delivery/ patients | Intervention subjects were significantly more likely to receive guideline-appropriate testing for cholesterol (OR = 1.39; [95%CI 1.07, 1.80] *p* = 0.012), creatinine (OR = 1.40; [95%CI 1.06, 1.84] *p* = 0.018), and proteinuria (OR = 1.74; [95%CI 1.13, 1.69] *p* = 0.012), but not A1C (OR = 1.17; [95% CI 0.80, 1.72] *p* = 0.43). Rates of control of A1C and LDL cholesterol were similar in the two groups. No differences in blood pressure, body mass index, or functional status were observed. | Vermont Diabetes Information System (VCIS) |
| Beaulieau et al. (2010) | Observational study | To develop a registry database linking administrative data to provide information of institution’s performance. | Patients undergoing cardiothoracic surgery (CABG and/or valve repair or replacement) | Through continuous feedback reduced transfusion rate by more than three standard deviations in the operative setting. | Clinical registry entered by a senior cardiovascular perfusionist linking with administrative data |
| Bricker et al. (2010) | Case study | To implement the Chronic Care Model and provide coaching, monthly measurement and patient registry support in order to improve performance. | Diabetes | Outcome measures as hemoglobin A1C, blood pressure and LDL cholesterol improved slowly over the years. Process improvements took place at a greater rate. | Electronic medical record system or an electronic patient registry |
| Bauer et al., (2011) | Quasi-experimental study | To analyze the role of site on specific process measures of depression treatment and on clinical outcomes by implementing a model of collaborative care | Patients having symptoms of depression between 2006 and 2009 | Among patients with who had valid PHQ-9 scores between six and 12 weeks the probability of discontinuing treatment differed significantly across clinics, as did the probability of improvement (range 0.36-0.84). Patients with early follow-up were more likely to improve (OR=1.64, *p*<0.01). | Web-based disease registry |
| Stern et al., (2011) | Observational study | To study how to realize continuous quality improvement through benchmarking. | Cystic fibrosis | A statistically significant correlation was found between the presence of Pseudomonas aeruginosa and reduced FEV1 (mean FEV1 98.9 vs. 87.3% in children and adolescents, 72.2 vs. 56.4% in adults, *p* = 0.001). | German Cystic Fibrosis Quality Assurance (CFQA) |
| Jakobsen et al., (2013) | observational study | To describe the methods used by DLCG and DLCR and the results obtained through this work. Also, to discuss possibilities how to improve the quality of lung cancer care through monitoring quality indicators. | All patients diagnosed in Denmark with primary lung cancer included in DLCR since Jan 1, 2000 through Dec 31, 2012 | One-year survival increased from 36.6% to 42.7%, 2-year survival has increased from 19.8% to 24.3%, 5-year survival from 9.8% to 12.1%. | Danish Lung Cancer Registry (DLCR) |
| Halpin et al. (2004) | Before-after study design | To describe how an interdisciplinary committee of health professionals led to a 50% reduction in the incidence of postoperative AF from 2000 to 2002. | All patients having CABG and valve replacement/repair from January 1, 2000 to June 30, 2002 | The incidence of postoperative atrial fibrillation for a CABG or valve replacement procedure decreased from 19% to 13.5%. Clinical pathway variances in length of stay secondary to atrial fibrillation decreased from 8.5% to 5.6%, which translates into a 1-2-day reduction in expected length of stay. | The STS National Adult Cardiac Surgery Database |
| Møller et al., (2005) | Registry-based cohort study | To develop and create methods for analysis and comparisons of pediatric-aged patients undergoing cardiac catherization and cardiac operation. | Pediatric-aged patients undergoing cardiac catherization and cardiac operation. | - Annual meetings of the participating hospitals were held. - Mortality for the centers has decreased from 12% (1982) to 6% (2001). - Center volume did not affect survival. - The data has been used to develop a consensus based method for risk adjustment. | The registry developed by the PCCC |
| Adams et al., (1998) | Prospective evaluation study | To determine if the outcome-based quality improvement model enhanced outcomes for health maintenance organization patients who receive care under contracted home health agencies. | Health maintenance organization patients referred for home health services who receive care from contracted home health agencies between April 29, 1996 and September 14, 1997. | The percentage of patients who improved between baseline and quarter four was not statistically significant. The percentages of stabilized patients on oral medications and on dyspnea were statistically significant greater at quarter four compared to baseline. | The Outcome Assessment and Information Set (OASIS) |
| Dziuban et al. (1994) | Report | To describe experiences of a New York State (NYS) cardiac surgery program. Methods by which staff used outcome data to discover meaningful information and program changes were studied | Cardiac surgery patients from in New York State the Department of Health (DOH) and specifically patients undergoing a CABG procedure | - Improvements resulted from a process of debating and searching and resulted in increased collaboration and sense of shared staff responsibility - Number deaths were reported in 1993 compared to 1992 and 1991 in patients undergoing emergency CABG procedure. Risk-adjusted mortality decreased compared to the public DOH data (3.7% in 1990, 6.6% in 1992, and 5.8% in 1992) 1.8% in 1993. Thus, internal results for 1993 indicate that overall actual mortality was about half of the 1992 data. | DOH data from New York State |
| Kraynack et al. (2009) | Observational study | To describe the process of gradual application of quality improvement methodology over 5 years by pediatric providers, at the Lewis Walker Cystic Fibrosis Center at Akron Children’s Hospital in Akron, Ohio. | Patient visiting CF clinic between the age of 6 to 18 years (for trial and later for all patients over the age of 6) | - A 5.9% relative increase in median FEV1 in the pilot of the pulmonary exacerbation score (PES). - Since standardization of PES median FEV1 has continued to improve in the 6-18 year old population. | The cystic fibrosis (CF) registry through the cystic fibrosis foundation (CFF) |
| Carlhed et al. (2009) | Before-after study design | To study a combination of a real-time, interactive  feedback generating national quality registry and a  systematic quality improvement collaborative on realizing clinical improvements. | Clinical Outcome After Acute Myocardial  Infarction | In the QUICC hospitals 2.8 lives per 100 patient years (14.2 to 11.4) and 9.3 readmissions for cardiac diagnoses per 100 patient years (49.5 to 40.2) were saved after comparing to before the QI intervention, corresponding to a 20% and 19% relative decrease in incidence. | RIKS-HIA |
| Thomas et al. (2007) | Randomized controlled trial | To implement registry-generated audit, feedback and patient reminders into an Internal Medicine (IM) resident continuity clinic and to assess the effect on process and intermediate outcomes | Diabetes | Clinical outcomes including HgbA1c, LDL cholesterol and blood pressure did not improve in the intervention group compared to the control group. | Clinical information systems, automatically queried clinical databases and reported summaries without manual effort |
| Lail et al., (2017) | Before-after study design | To help disease-based teams use the principles of improvement science and implement components of the CCM. To improve care for children with chronic and complex conditions. | Children with chronic conditions. | 50% of included patients had the desired or an improved outcome. 25% had improvement in disease remission higher than expected, 3% improvement higher than expected in disease control, 21% improvement higher than expected in quality of life, 20% improvement higher than expected in symptom management. Eleven of the 18 participating teams achieved the goal of 20% improvement in their chosen outcome. | Electronic health record of the Cincinnati Children’s Hospital Medical |
| Baty et al. (2010) | Observational study | To study whether the application of a successful system-based approach making use of a computerized patient registry could reduce disparity in care for cultural, ethnic and socioeconomic minorities | Diabetes | Every tracked indicator improved except HbA1c control > 9%. Mean outcomes in diabetes quality measures ranged from +22% improvement in the percent of patients with HbA1c below 7% to +400% mean improvement in the percent of patients who had a retinopathy screen in the past year. | Expanded patient registry used at Advantage Health physician offices (CDEMS) |
| Toh et al., (2009) | Report | To facilitate continuity of care for patients with chronic diseases and for greater efficiency in outcome management. | Diabetes | There was a gradual reduction of patients with poor HbA1c (9% and above) in primary care clinic patients from 12% to 9% and an increase in the proportion of patients with good LDL-c control from 35.5% to 52.0%. At the hospitals a similar trend in LDL-c control was observed, but the proportion of patients with poor HbA1c remained the same. | Chronic disease management registry |
| Siracusa et al. (2013) | Report | To improve clinical outcomes (FEV1 and BMI) for patients with CF through quality improvement aimed at increasing patient centeredness and improving healthcare delivery | Patients with cystic fibrosis age 0-21 | Median FEV1 increased from 81.7% to 100.1% with an absolute improvement of 18.4% predicted. BMI improved from the 35^th^ centile to the 55^th^ centile, which was a 1.7-fold improvement. | The Cystic Fibrosis Foundation National Patient Registry |
| Han et al. (2016) | Observational study | To examine the impact of using a registry for patient reminders is associated with differences in quality of care and hospital utilization rates | Diabetes | Patients with type 2 diabetes trated in practices using registries for patient reminders were more likely to have completed the recommended laboratory testing (OR=1.26, *p*<0.01) and dilated retinal examinations (OR=1.14, *p*<0.01). Patients in practices with registries for quality improvement were less likely to have avoidable hospitalization (OR=9.83, *p*<0.01) and emergency room visits (OR=0.76, *p*<0.01). There was no effect of the use of a diabetes registry on quality of care for patients with type 1 diabetes | Data obtained from electronic health record system and claims data |

**Appendix 5a.** Scoring of the Downs & Black criteria, SQUIRE guidelines and additional self-developed tool.

|  | Stern et al. (2011) | Peterson et al. (2015) | Peterson et al. (2008) | Adams et al. (1998) | Halpin et al. (2004) | Moller et al. (2005) | Thomas et al. (2007) | MacLean et al. (2009) | Kraynack & MacBride (2009) | Beaulieau et al (2010) |
| --- | --- | --- | --- | --- | --- | --- | --- | --- | --- | --- |
|  | Downs & Black criteria ^a^ | | | | | | | | | |
| Have the main outcomes to be measured been clearly described in the introduction or methods section? (2) | N | Y | Y | N | Y | Y | Y | Y | Y | N |
| Were the statistical tests used to assess the main outcomes appropriate? (18) | N | Y | Y | Y | Y | Y | Y | Y | Y | Y |
| *Total score (Y)* | **0** | **2** | **2** | **1** | **2** | **2** | **2** | **2** | **2** | **1** |
|  | Squire Guideline ^b^ | | | | | | | | | |
| Was a method employed for assessing completeness and accuracy of data? (10c) | Y | N | N | N | N | N | N | N | N | Y |
| Were quantitative methods used to draw inferences from the data?* (11a) | Y | Y | N | Y | Y | Y | Y | Y | Y | Y |
| Were methods applied for understanding variation within the data, including the effects of time as a variable? (11b) | N | N | N | N | N | N | N | N | N | N |
| *Total score (Y)* | **2** | **1** | **0** | **1** | 1 | **1** | 1 | **1** | 1 | **2** |
|  | Self-developed Checklist | | | | | | | | | |
| Has a method been applied for handling missing values?*Remarks: What is the threshold that was applied? | N | N | Y | N | N | N | N | Y | N | N |
| Has an audit / data check been performed? *Remarks: Sanity checks on logical correlations between variables? Outliers? Etc. | N | N | N | N | N | N | N | N | N | Y |
| Do the researchers discuss secular trends (trends in data due to improvements in health care over time independent to the study)? | N | Y | N | N | N | N | N | N | N | N |
| Do the researchers discuss the impact of other changes/QI processes within the hospital potentially interfering with outcomes? | N | Y | N | N | N | N | N | N | N | N |
| Have outcomes in the analysis been adjusted for case mix? Remarks: If not, is it discussed why? Etc. | N | N | Y | Y | N | Y | N | Y | N | N |
| Are definitions given for the main outcomes (or references to those definitions)? | Y | Y | Y | N | Y | Y | Y | N | Y | N |
| Is the patient group/target group of the registry described?*Remarks: Are clear definitions given including inclusions and exclusions? | Y | Y | Y | Y | Y | Y | Y | Y | Y | Y |
| Has a power analysis been conducted? | N | N | Y | N | N | N | Y | Y | N | N |
| *Total score (Y)* | **2** | **4** | **5** | **2** | ***2*** | **3** | **3** | **4** | **2** | **2** |

^a^ From the Downs & Black questionnaire, question 2 and 18 have been used (Downs & Black 1998).

^b^ From the SQUIRE guidelines, question 10c,11a and 11b have been used (Ogrinc et al., 2008).

**Appendix 5b.** Scoring of the Downs & Black criteria, SQUIRE guidelines and additional self-developed tool.

|  | Bricker et al. (2010) | Jakobsen et al., (2013) | Jacobsen et al., (2009) | Dziuban et al., (1994) | Carlhed et al., (2008) | Han et al. (2016) | Baty et al. (2010) | Bauer et al. (2011) | Siracusa et al. (2014) | Lail et al., (2017) | | Toh et al., (2009) |
| --- | --- | --- | --- | --- | --- | --- | --- | --- | --- | --- | --- | --- |
|  | Downs & Black criteria ^a^ | | | | | | | | | | | |
| Have the main outcomes to be measured been clearly described in the introduction or methods section? (2) | N | Y | Y | Y | Y | Y | Y | Y | Y | Y | | Y |
| Were the statistical tests used to assess the main outcomes appropriate? (18) | Y | N | Y | Y | Y | Y | Y | Y | N | Y | | N |
| *Total score (Y)* | **1** | 1 | **2** | **2** | **2** | **2** | **2** | **2** | 1 | **2** | | 1 |
|  | Squire Guideline ^b^ | | | | | | | | | | | |
| Was a method employed for assessing completeness and accuracy of data? (10c) | N | Y | N | Y | Y | N | N | N | N | N | | N |
| Were quantitative methods used to draw inferences from the data?* (11a) | Y | Y | Y | Y | Y | Y | Y | Y | Y | Y | | N |
| Were methods applied for understanding variation within the data, including the effects of time as a variable? (11b) | N | N | N | Y | Y | N | N | Y | N | N | | N |
| *Total score (Y)* | **1** | **2** | **1** | **3** | **3** | 1 | **1** | 3 | 2 | **1** | | **0** |
|  | Self-developed Checklist | | | | | | | | | | | |
| Has a method been applied for handling missing values?*Remarks: What is the threshold that was applied? | N | N | N | N | N | N | N | N | N | | N | N |
| Has an audit / data check been performed? *Remarks: Sanity checks on logical correlations between variables? Outliers? Etc. | N | Y | N | N | Y | N | N | N | N | | N | N |
| Do the researchers discuss secular trends (trends in data due to improvements in health care over time independent to the study)? | N | N | Y | N | Y | N | N | N | N | | N | N |
| Do the researchers discuss the impact of other changes/QI processes within the hospital potentially interfering with outcomes? | N | N | N | N | Y | N | N | N | N | | N | Y |
| Have outcomes in the analysis been adjusted for case mix? Remarks: If not, is it discussed why? Etc. | N | N | N | N | Y | Y | N | Y | N | | N | N |
| Are definitions given for the main outcomes (or references to those definitions)? | N | Y | Y | Y | Y | Y | Y | Y | Y | | Y | Y |
| Is the patient group/target group of the registry described?*Remarks: Are clear definitions given including inclusions and exclusions? | Y | Y | Y | Y | Y | Y | Y | Y | Y | | Y | Y |
| Has a power analysis been conducted? | N | N | N | N | N | N | N | N | N | | N | N |
| *Total score (Y)* | **1** | 3 | **3** | **3** | **6** | **3** | **2** | **3** | 2 | | 2 | 3 |

^a^ From the Downs & Black questionnaire, question 2 and 18 have been used (Downs & Black 1998).

^b^ From the SQUIRE guidelines, question 10c,11a and 11b have been used (Ogrinc et al., 2008).
